# A pioneering *RET* genetic screening study in the State of Ceará, Brazil, evaluating patients with medullary thyroid cancer and at-risk relatives: experience with 247 individuals

**DOI:** 10.20945/2359-3997000000088

**Published:** 2018-10-01

**Authors:** Maria Cecília Martins-Costa, Susan C. Lindsey, Lucas L. Cunha, Fernando Porto Carreiro-Filho, André P. Cortez, Marcelo E. Holanda, J. Wilson M. de Farias, Sérgio B. Lima, Luís A. Albano Ferreira, Pedro Collares Maia, Cléber P. Camacho, Gilberto K. Furuzawa, Ilda S. Kunii, Magnus R. Dias-da-Silva, João R. M. Martins, Rui M. B. Maciel

**Affiliations:** 1 Universidade Federal de São Paulo Universidade Federal de São Paulo Escola Paulista de Medicina Departamento de Medicina São Paulo SP Brasil Centro de Doenças da Tiroide e Laboratório de Endocrinologia Molecular e Translacional, Divisão de Endocrinologia, Departamento de Medicina, Escola Paulista de Medicina, Universidade Federal de São Paulo (EPM-Unifesp), São Paulo, SP, Brasil; 2 Hospital Geral de Fortaleza Hospital Geral de Fortaleza Centro de Endocrinologia e Metabologia Fortaleza CE Brasil Centro de Endocrinologia e Metabologia, Hospital Geral de Fortaleza (HGF), Fortaleza, CE, Brasil; 3 Universidade de Fortaleza Universidade de Fortaleza Departamento de Medicina Fortaleza CE Brasil Departamento de Medicina, Universidade de Fortaleza (UNIFOR), Fortaleza, CE, Brasil; 4 Hospital Geral de Fortaleza Hospital Geral de Fortaleza Departamento de Cirurgia de Cabeça e Pescoço Fortaleza CE Brasil Departamento de Cirurgia de Cabeça e Pescoço, Hospital Geral de Fortaleza, Fortaleza, CE, Brasil; 5 Santa Casa de Misericórdia de Fortaleza Santa Casa de Misericórdia de Fortaleza Fortaleza CE Brasil Santa Casa de Misericórdia de Fortaleza, Fortaleza, CE, Brasil; 6 Hospital Geral Dr. César Cals Fortaleza CE Brasil Hospital Geral Dr. César Cals, Fortaleza, CE, Brasil; 7 Universidade Federal do Ceará Universidade Federal do Ceará Hospital Universitário Walter Cantídio Fortaleza CE Brasil Hospital Universitário Walter Cantídio, Universidade Federal do Ceará (UFC), Fortaleza, CE, Brasil; 8 Hospital Infantil Albert Sabin Fortaleza CE Brasil Hospital Infantil Albert Sabin, Fortaleza, CE, Brasil; 9 Centro Universitário Christus Centro Universitário Christus Fortaleza CE Brasil Centro Universitário Christus (Unichristus), Fortaleza, CE, Brasil; 10 Universidade Federal de São Paulo Universidade Federal de São Paulo Escola Paulista de Medicina Departamento de Bioquímica São Paulo SP Brasil Divisão de Biologia Molecular, Departamento de Bioquímica, Escola Paulista de Medicina, Universidade Federal de São Paulo (EPM-Unifesp), São Paulo, SP, Brasil; 11 Fleury Medicina e Saúde Fleury Medicina e Saúde São Paulo SP Brasil Fleury Medicina e Saúde, São Paulo, SP, Brasil

**Keywords:** Medullary thyroid carcinoma, *RET* mutation, genetic screening

## Abstract

**Objective:**

Initial diagnosis of medullary thyroid carcinoma (MTC) is frequently associated with advanced stages and a poor prognosis. Thus, the need for earlier diagnoses and detection in relatives at risk for the disease has led to increased use of *RET* genetic screening.

**Subjects and methods:**

We performed *RET* screening in 247 subjects who were referred to the Brazilian Research Consortium for Multiple Endocrine Neoplasia (BRASMEN) Center in the State of Ceará. Direct genetic sequencing was used to analyze exons 8, 10, 11, and 13-16 in MTC index cases and specific exons in at risk relatives. Afterward, clinical follow-up was offered to all the patients with MTC and their affected relatives.

**Results:**

*RET* screening was performed in 60 MTC index patients and 187 at-risk family members. At the initial clinical assessment of the index patients, 54 (90%) were diagnosed with apparently sporadic disease and 6 (10%) diagnosed with hereditary disease. After *RET* screening, we found that 31 (52%) index patients had sporadic disease, and 29 (48%) had hereditary disease. Regarding at-risk relatives, 73/187 were mutation carriers. Mutations in *RET* codon 804 and the rare p.M918V mutation were the most prevalent.

**Conclusions:**

Performing *RET* screening in Ceará allowed us to identify a different mutation profile in this region compared with other areas. *RET* screening also enabled the diagnosis of a significant number of hereditary MTC patients who were initially classified as sporadic disease patients and benefited their relatives, who were unaware of the risks and the consequences of bearing a *RET* mutation.

## INTRODUCTION

Medullary thyroid carcinoma (MTC) is an uncommon malignant tumor arising from the calcitonin-producing parafollicular cells (C-cells) of the thyroid and accounts for 1-2% of all thyroid cancers in the US (1). Regarding the Brazilian population, the latest estimates indicate that 5,870 and 1,090 new cases of all types of thyroid cancer have been diagnosed among women and men, respectively, in 2016 (2). Regarding Ceará, a State of the Northeastern region of Brazil with an estimated population of 9,107,101 inhabitants, the latest estimates indicate that 460 and 100 new cases of all types of thyroid cancer have been diagnosed among women and men, respectively (2).

In a Brazilian study about thyroid cancer incidence patterns in the State of São Paulo from 1997-2008, Veiga and cols. noted incidences of MTC of 0.56 per 100,000 persons in women and 0.15 per 100,000 persons in men (3). Assuming that the incidence of this neoplasm in Ceará is similar to that reported in the State of São Paulo, approximately 26 and 7 new cases of MTC would be diagnosed in women and men, respectively, per year (3).

The sporadic form of MTC is the most common presentation of MTC (approximately 75% of cases) (4), but in 25% of cases, MTC may also occur as part of an inherited disorder referred to as multiple endocrine neoplasia type 2 (MEN 2). MEN 2 is caused by germline mutations in the *RE*arranged during *T*ransfection (*RET*) gene and is transmitted as an autosomal dominant trait with variable degrees of expressivity and age-related penetrance (5).

The clinical diagnosis of MTC by palpation of a thyroid nodule or mass on physical examination is usually associated with advanced TNM stages and a poor prognosis. In this context, *RET* molecular analysis assumes vital importance, as this analysis allows earlier diagnosis of MTC, thereby increasing the chance of curative treatment (1). Additionally, *RET* molecular analysis enables the diagnosis of hereditary MTC in some apparently sporadic cases, which favors more careful management of other diseases that may accompany MEN 2 syndrome.

This study aimed to report the clinical pattern of MTC in the State of Ceará and to describe the molecular profile of this neoplasm using *RET* gene analysis.

## SUBJECTS AND METHODS

### Subjects

We report the results of our experience with *RET* genetic screening of patients with a diagnosis of MTC, as well as their at-risk relatives, who were evaluated at a single center in Ceará, Brazil, from May 2009 to December 2015. This center is located in the city of Fortaleza, the capital of Ceará, and is part of a Brazilian Research Consortium for Multiple Endocrine Neoplasia (BRASMEN). All *RET* sequencings performed in this study were conducted in the Laboratory of Molecular and Translational Endocrinology, Division of Endocrinology, Department of Medicine, Escola Paulista de Medicina, Universidade Federal de São Paulo (EPM/Unifesp).

All subjects investigated in this study signed informed consent. The study protocol was approved by the local internal review board (CAAE: 16441414.9.1001.5505111).

The patients included in this study had histopathologically confirmed diagnoses of MTC. Immunohistochemical studies for calcitonin, synaptophysin, and chromogranin were performed when histopathological exams were inconclusive, and no patients presented with elevated serum calcitonin (sCt) or CEA levels. All the patients were originally from Ceará. The exclusion criteria were: patients from other countries or other Brazilian States, relatives of deceased MTC index patients without available histopathologic information to confirm their relative's MTC diagnosis and patients with uncertain histopathology.

### Clinical evaluation before *RET* screening

Patients initially underwent a clinical assessment and genetic counseling before undergoing peripheral blood collection for *RET* analysis. Patients were warned about the risks of having additional endocrine diseases related to MEN 2 if a *RET* mutation was identified, as well as the risks facing their relatives bearing the same mutation. Therefore, a portion of the patient population analyzed in this study comprised the at-risk relatives of patients diagnosed with hereditary MTC after *RET* sequencing.

In addition, a questionnaire was designed to acquire the following general information about each patient: age upon diagnosis of MTC/CCH and upon *RET* screening; previous knowledge of MTC/CCH upon total thyroidectomy (TT), if previously performed; preoperative cervical ultrasound or thyroid nodule or lymph node (LN) fine-needle aspiration biopsy (FNAB) findings; sCt levels; and the referring doctor's specialty.

### *RET* mutation analysis

Genomic DNA was extracted from peripheral blood leucocytes using an in-house protocol (6). Sequence analysis of hot-spot-bearing exons 8, 10, 11, and 13-16 was performed, and extended *RET* gene analysis was also conducted (7) in all patients considered to have sporadic disease according to hot-spot exon 8, 10, 11, 13-16 analysis who presented with multifocal MTC or were young and experienced an unfavorable follow-up course (7).

### Clinical evaluation and follow-up after *RET* screening

In agreement with their physicians, patients with a diagnosis of MTC and the mutation-carrier relatives were subjected to further biochemical and imaging evaluations. Some of the patients continued to be followed up in the Ceará BRASMEN Center.

Patients with MTC who had already undergone surgical treatment at the time of *RET* genetic screening were initially followed with sCt, CEA, ionized calcium, TSH, and cervical US assessments every 4 months. Imaging procedures to detect metastases, such as abdominal magnetic resonance (MRI) or 3-phase contrast-enhanced multidetector liver CT, thoracic CT, column and sacral MRI and bone scans, were performed whenever sCt levels were higher than 150 pg/mL. Patients with hereditary MTC and their mutation-carrier relatives who had already undergone surgical treatment were followed with the same complementary tests, as well as annual 24-hour urine catecholamine and metanephrine assessments. Patients who were diagnosed with MTC after *RET* screening were encouraged to undergo specific surgical treatments according to their preoperative sCt and imaging results, and mutation carriers were encouraged to undergo additional evaluations to screen for PHEO, HPTH, and other MEN 2 syndrome components.

### Histopathology

The histological diagnosis of MTC was rendered according to the 2015 ATA guidelines (1), according to the presence of the basic histological pattern of the disease (*i.e.,* typically round, polyhedral, or spindle-shaped and formed sheets or nests, with peripheral palisading in the vascular stroma, and the presence of stromal amyloid) and the typical immunohistochemical (cells positive for calcitonin, synaptophysin and chromogranin) findings.

## STATISTICAL ANALYSIS

The results are presented as the mean ± standard deviation (SD) and as ranges (minimum-maximum). Mann-Whitney U tests and analyses of variance (ANOVA) were used to compare quantitative variables between two and three or more groups, respectively, and Fisher's exact test was used to analyze changes in categorical variables. SPSS software (version 23; SPSS Inc., Chicago, IL, USA) was used to perform the analysis. Statistical significance was indicated by p < 0.05.

## RESULTS

### Patients and relatives

Overall, over the last 5 years, we performed 263 *RET* sequencing tests in the BRASMEN-Ceará study. Sixteen patients met one or more of the exclusion criteria described above. Therefore, we performed *RET* genetic screening in 247 subjects, including 60 index patients with MTC and 187 at-risk relatives, from the State of Ceará, Brazil. Head-and-neck surgeons referred most of the index patients (Table 1).

**Table 1.** Preoperative clinical data and *RET* screening results for the index patients with sporadic and hereditary disease and *RET* mutation-carrier relatives with confirmed diagnoses of MTC/CCH after total thyroidectomy

Clinical data	Index		Relatives^b^		*p*
	Sporadic (n = 31)	Hereditary (n = 28)^a^	Before *RET* screening (n = 6)	After *RET* screening (n = 18)	
**Gender (Male:Female)**	7:24	7:21	1:5	7:11	< 0.05^e^
**Age at *RET* screening (years)**	n = 31 (100%)	n = 28 (100%)	n = 6 (100%)	n = 18 (100%)	
Mean ± SD	50 ± 13	48 ± 15	52 ± 23	36 ± 21	NS
Median (range)	50 (22 – 37)	52 (16 – 71)	49 (29 – 93)	32 (7 – 77)	
***RET* screening**	n = 31 (100%)	n = 28 (100%)	n = 6 (100%)	n = 18 (100%)	
Before TT	1	0	6	0	
After TT	30	28	0	18	
**Age at diagnosis of MTC/CCH (years)**	n = 31 (100%)	n = 28 (100%)	n = 6 (100%)	n = 18 (100%)	
Mean ± SD	48 ± 15	43 ± 14	42 ± 17	37 ± 21	NS
Median (range)	50 (20 – 73)	42 (16 – 65)	48 (17 – 57)	33 (8 – 77)	
**Previous knowledge of MTC/CCH diagnosis at TT**	n = 31 (100%)	n = 28 (100%)	n = 6 (100%)	n = 18 (100%)	
Not known	18	22	6	9	< 0.05^f^
By LN biopsy	2	1	0	0	
By thyroid nodule/LN FNAB cytology and level of sCt > 100 pg/mL	10	5	0	2	
Probable (sCt > 100 pg/mL without + cytology for MTC)	1	0	0	7	
**Characteristics of preoperative US**^c^	n = 31 (100%)	n = 28 (100%)	n = 6 (100%)	n = 18 (100%)	
Without thyroid nodules at US	0	0	0	1	
Not available	12	12	2	0	
Suspicious thyroid nodule at US	14	10	4	10	NS
Non-suspicious thyroid nodule at US	5	6	0	7	
**Preoperative cytology**	n = 24 (77%)	n = 17 (61%)	n = 4 (67%)	n = 11 (61%)	
Not performed/Result not available	6 / 1	8 / 4	1 / 1	7 / 0	
Insufficient	0	1	0	0	
Insular neoplasia	1	0	0	0	
Benign: paucicellular/satisfactory cellularity	1 / 6	2 / 2	2 / 1	0 / 2	
Atypia of indeterminate significance	1	0	0	5	
Follicular	4	4	1	0	
Suggestive of PTC/PTC	0	3	0	2	
Suggestive of MTC/MTC	10	5	0	2	NS
Suggestive of anaplastic carcinoma	1	0	0	0	
**Preoperative sCt (pg/mL)**^d^	n = 8 (26%)	n = 5 (18%)	n = 0	n = 17 (94%)	
Mean ± SD	855 ± 1389	1266 ± 1929	-	343 ± 709	NS
Median (range)	159 (19-4020)	347 (12-4640)	-	98 (8-2695)	
**Reference for *RET* screening**	n = 31 (100%)	n = 28 (100%)	n = 6 (100%)	n = 18 (100%)	
Endocrinologist	9	5	1	0	
Head and Neck surgeon	22	21	3	1	
General surgeon	0	1	0	0	
Oncologist	0	1	0	0	
Family member	0	0	2	17	

MTC: medullary thyroid cancer; CCH: C-cell hyperplasia; SD: standard deviation; TT: total thyroidectomy; LN: lymph node; FNAB: fine-needle aspiration biopsy; sCt: serum calcitonin; US: ultrasound; PTC: papillary thyroid cancer.

a One index patient was lost to follow-up before TT.

b One mutation-carrier relative did not undergo TT.

c Nodules were considered suspicious if they exhibited one of the following characteristics: size greater than 3 cm, microcalcifications, or central flow on Doppler US, and suspected lymph node.

d Cut-off values of 18.4 pg/mL for males and 7.8 pg/mL for females.

e Fisher's exact test.

f Not known *versus* known (Fisher's exact test)

**Figure 1:**
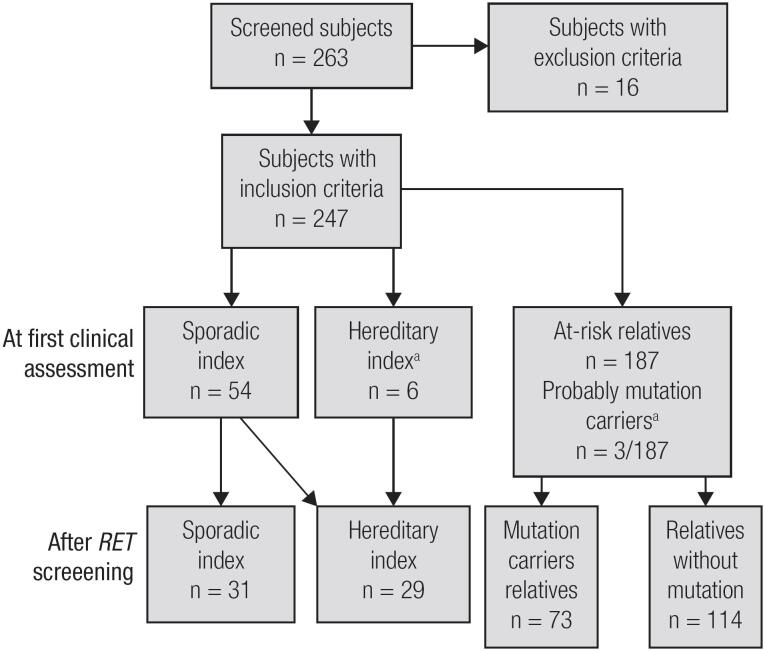
Fluxogram of the study according to the first clinical assessment or *RET* screening. aProbable *RET* mutation carriers identified before genetic screening due to either a personal history of MEN 2 or physical examination findings suggestive of MEN 2.

During the initial phase of the study, based only on *RET*-screened patient clinical history and physical examination results, we identified only 6 index patients and three relatives with hereditary MTC. These patients were characterized as hereditary index patients because they had another family member with MTC or a personal/familial history of MEN 2. In the second phase of the study, after performing *RET* sequencing, we observed that 23 MTC index patients who were initially classified as sporadic disease patients were, in fact, hereditary index patients. Regarding at-risk *RET*-screened relatives, we found that 73 relatives carried mutations, and 114 relatives were non-carriers (Figure 1 and Table 2). The clinical and genetic data of the MTC index patients and the mutation-carrier relatives are shown in Table 2.

**Table 2:** Genetic and clinical features of the 29 patients considered index cases upon *RET* sequencing

Index cases	Age at *RET* sequencing (years)	Age at MTC diagnosis (years)	Relatives affected by MTC at time of screening	Exon	*RET* mutation	Phenotype	Months of follow up	Mutation carriers/screened relatives	Relatives carrying mutation with MTC/CCH^a^	
									Before *RET* screening	After *RET* screening
1^b^	58	59	0	10	p.C609G	MTC	10	0/2	0	0
2^b^	62	30	0	10	p.C611R	MTC	36	0	0	0
3^b^	35	34	0	10	p.C630R	MTC	29	1/6	0	1
4	26	22	3	11	p.C634R	MTC + PHEO + CLA	47	6/13	2	3
5^b^	22	21	0	11	p.C634R	MTC	14	1/2	0	0
6	43	37	0	11	p.C634R	MTC + PHEO	2	0	0	0
7	40	20	0	11	p.C634R	MTC + PHEO + CLA	42	1/4	0	1
8^b^	41	41	0	13	p.L790F	MTC	16	0	0	0
9^b^	63	61	0	14	p.V804L	MTC	21	5/10	0	2
10^b^	51	39	0	14	p.V804L	MTC	10	5/10	0	0
11^b^	54	54	0	14	p.V804L	MTC	3	0	0	0
12	67	56	1	14	p.V804L	MTC	4	1/1	1	0
13^b^	42	42	0	14	p.V804L	MTC	69	1/3	0	0
14^b^	52	49	0	14	p.V804L	MTC	59	3/6	0	2
15^b^	71	65	0	14	p.V804L	MTC	8	0/1	0	0
16^b^	61	55	0	14	p.V804M	MTC	8	1/1	0	1
17	45	41	1	14	p.V804M	MTC	3	2/3	0	0
18^b^	43	43	0	14	p.V804M	MTC	5	2/2	0	0
19^b^	36	36	0	15	p.S891A	MTC	76	1/4	0	0
20^b^	54	54	0	16	p.M918V	MTC	67	33/95	3	7
21^b^	59	58	0	16	p.M918V	MTC	21	0/0	0	0
22^b^	55	55	0	16	p.M918V	MTC	70	1/5	0	0
23^b^	42	40	0	16	p.M918V	MTC	70	4/7	0	1
24^b^	66	59	0	16	p.M918V	MTC	71	3/3	0	0
25^b^	28	24	0	16	p.M918V	MTC	74	1/3	0	1
26^b^	55	55	0	16	p.M918V	MTC	51	0/0	0	0
27^b^	54	40	0	16	p.M918V	MTC	52	0/0	0	0
28^b^	69	58	0	16	p.M918V	MTC	15	1/4	0	0
29	16	16	0	16	p.M918T	MEN 2B	1	0/2	0	0
TOTAL (mean ± SD)	48.6 ± 14.4	43.6 ± 14.1^c^	5	--	--	--	32.9 ± 27.3	73/187 (39%)	6	19
Median (range)	52 (16-71)	42 (16-65)	--	--	--	--	21 (1-76)	--	--	--

*RET*: REarranged during *T*ransfection protooncogene; MTC: medullary thyroid cancer; CCH: C-cell hyperplasia; PHEO: pheochromocytoma; CLA: cutaneous lichen amyloidosis; HPTH: primary hyperparathyroidism; SD: standard deviation.

a This column refers only to relatives who underwent *RET* screening. Relatives who probably carry *RET* mutations (by clinical assessment only) but did not undergo *RET* screening were not included. Diagnoses of MTC/CCH were made based on histopathology results after TT (n = 18) or when relatives who had not undergone TT had fine-needle aspiration cytology positive for MTC concurrently with elevated serum calcitonin levels (above 100 pg/mL) (n = 1).

b Patients with apparent sporadic disease based on their clinical assessment and *RET* screening results.

c Not significant (Mann-Whitney U test).

**Figure 2:**
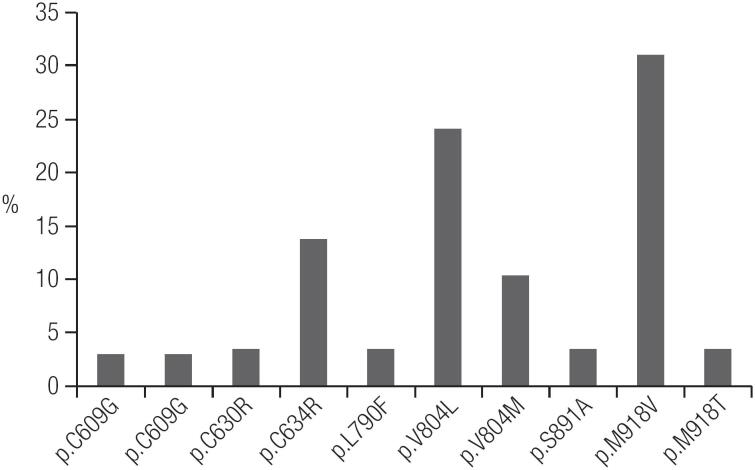
Profiles of *RET* gene mutations in Ceará.

The mutation profile of *RET* mutations found in Ceará are shown in Figure 2. Overall, of the 29 hereditary MTC index patients who underwent *RET* screening, 28 definitely underwent TT, and the remaining patient was lost to follow-up (Table 2). In all but one of the MTC index patients, *RET* screening was performed only postoperatively (Table 1).

Regarding the mutation-carrier relatives, 25 patients presented MTC/CCH confirmed by either histopathology after TT (n = 24) or by FNAB cytology positive for MTC along with elevated sCt levels above 100 pg/mL (n = 1) (Table 2). Among those 24 patients with positive histopathology, 6 had already been diagnosed with MTC before *RET* screening, as they underwent evaluations for asymptomatic thyroid nodules because they knew about index cases in their families (2 relatives of index patient #4, 1 relative of index patient #12) or because they noticed thyroid nodule growth but were not aware that they had a family member with MTC until a broad pedigree was performed (3 relatives of index patient #20, Table 2). In the remaining patients (n = 18), *RET* screening led to the diagnosis of MTC/CCH (Tables 1 and 2). In 2 of these 18 patients, we found only CCH. Only one mutation-carrier relative referred to TT had neither MTC nor CCH on histopathology. She was a p.V804L mutation carrier and had undergone TT at 9 years of age.

### Genotype-phenotype correlation

Based on the clinical evaluation and *RET* genetic screening results, we identified 29 index patients with hereditary MTC and 31 index patients with sporadic MTC (Figure 1). The most frequent mutations found were p.V804L and p.M918V (Table 2, Figure 2). PHEO was associated with the following mutations: p.C634R, p.V804L, and p.M918T. HPTH was associated with p.C634R and p.V804L, and LCA was associated with p.C634R. There were no cases of MEN 2A with HD (Table 2). Patients with the p.M918B mutation did not present the MEN 2B phenotype (8). We identified only one patient with MEN 2B, who had the classical p.M918V mutation.

Unsuspected germline *RET* mutations were found in 23 of the 54 MTC patients (43%) who presented with apparent sporadic disease, according to their negative familial histories and MTC-only phenotypes. These patients were informed about the hereditary origins of their neoplasms, and *RET* genetic screening was subsequently offered to their relatives (Figure 1).

### Preoperative evaluation of *RET*-screened patients

The preoperative data of the *RET*-screened patients who underwent TT are shown in Table 1. The majority of patients were women. Additionally, the mutation-carrier relatives whose neoplasm diagnoses were made after *RET* screening were younger than the patients whose neoplasm diagnoses were made before genetic analysis (not significant).

**Figure 3:**
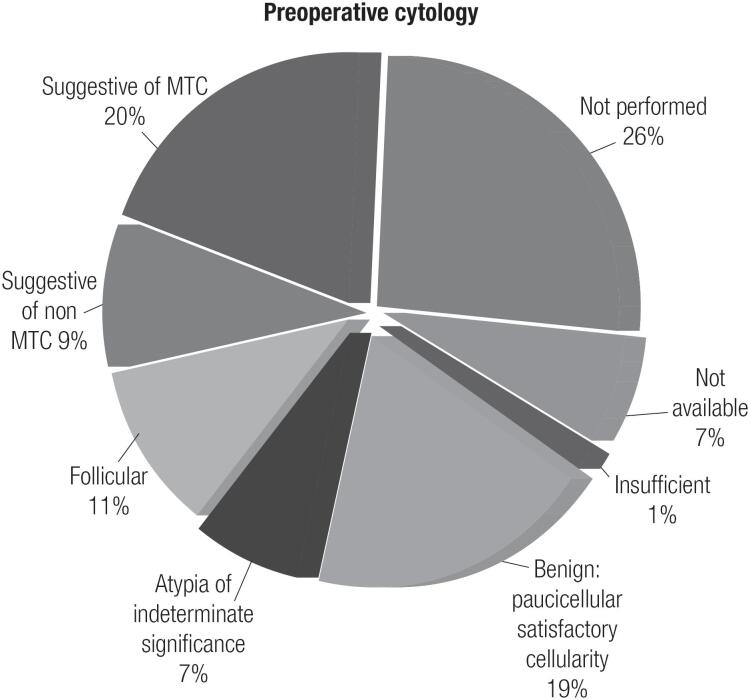
Preoperative cytology of index patients with sporadic and hereditary disease and their *RET* mutation-carrier relatives.

In most patients, MTC/CCH was diagnosed based on histopathological findings following TT because only 20% of patients had cytology results suggestive of MTC (Figure 3), and consequently, only a few patients had preoperative sCt measurements. However, as expected, we observed lower levels of sCt in mutation-carrier relatives than in hereditary index patients, although the difference in tumor marker levels between the two groups was not statistically significant (Table 1).

In 9 of the 18 mutation-carrier relatives diagnosed with MTC/CCH after *RET* screening, diagnoses for MTC had already been made possible by their clinical evaluations, as they had sCt levels above 100 pg/mL along with a suspicious thyroid nodule on US and/or FNAB thyroid nodule or cervical LN cytology results that were suspicious for malignancy (Table 1).

### Postoperative evaluation

**Figure 4:**
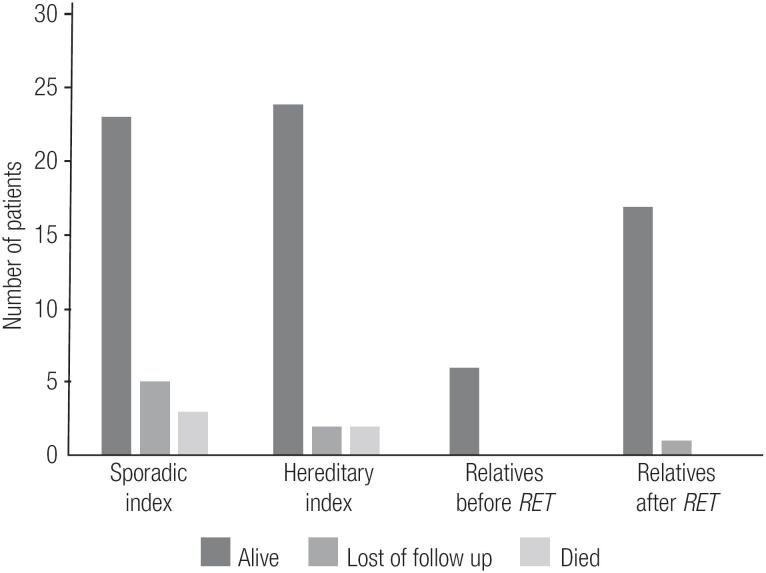
Current follow-up status of index (sporadic and hereditary) patients and *RET*-carriers relatives who underwent total thyroidectomy.

The postoperative data for the *RET*-screened patients who underwent TT and received a confirmed diagnosis of MTC are shown in Tables 3 and 4. An interesting finding was that relatives whose diagnoses of MTC/CCH were made after *RET* screening had lower sCt levels and less frequent postsurgical hypoparathyroidism than index patients and mutation-carrier relatives whose diagnoses of MTC/CCH were made before *RET* screening (p < 0.05) (Table 3). Also, up to the most current follow-up, all relatives diagnosed with MTC/CCH after *RET* screening were alive, while 5 index cases had died (Figure 4, Table 3).

**Table 3:** Postoperative clinical data for the index patients with sporadic and hereditary disease and *RET* mutation-carrier relatives with confirmed MTC/CCH after total thyroidectomy

Clinical data	Index		Relatives		*p*
	Sporadic (n = 31)	Hereditary (n = 28)	Before *RET* screening (n = 6)	After *RET* screening (n = 18)	
**Age at TT (years)**	n = 31 (100%)	n = 28 (100%)	n = 6 (100%)	n = 18 (100%)	
Mean ± SD	47 ± 15	43 ± 14	41 ± 16	35 ± 18	NS
Median (range)	48 (20-73)	42 (16-65)	48 (17-57)	33 (8-67)	
**Clinical presentation** a **At diagnosis/at last medical visit**	n = 31 (100%)	n = 28 (100%)	n = 6 (100%)	n = 18 (100%)	
Without LN and/or systemic metastases	22 / 21	14 / 12	3 / 0	13 / 13	NS
Central LN metastases	0 / 0	5 / 0	1 / 0	2 / 2	
Lateral LN metastases	8 / 4	7 / 12	2 / 5	3 / 3	
Systemic metastases	1 / 6	2 / 4	0 / 1	0 / 0	
**EBRT**	n = 31 (100%)	n = 28 (100%)	n = 6 (100%)	n = 0	
Performed	6	7	1	0	NS
Not performed	25	21	5	0	
**Post-surgical hypoparathyroidism**^b^	n = 30 (97%)	n = 28 (100%)	n = 6 (100%)	n = 18 (100%)	
Yes	7	14	0	1	< 0.05^c^
No	23	14	6	17	
**Last serum Ct of follow up (pg/mL)**	n = 27 (87%)	n = 22 (79%)	n = 6 (100%)	17 (94%)	
Mean ± SD	437 ± 1223	324 ± 890	192 ± 317	26 ± 71	< 0.05^d^
Median (range)	2 (2-5660)	7 (2-3860)	43 (8-818)	2 (2-273)	
**Current follow up**	n = 26 (94%)	n = 24 (86%)	n = 6 (100%)	n = 17 (94%)	
At Brasmen-Ceará Center Only	4	3	0	5	
At Brasmen-Ceará and original doctor	10	14	5	8	
At original doctor only	9	7	1	4	
Lost of follow up	5	2	0	1	
Died	3	2	0	0	
**Time of follow-up (months)**	n = 31 (100%)	n = 28 (100%)	n = 6 (100%)	n = 18 (100%)	
Mean ± SD	19 ± 22	35 ± 27	36 ± 18	40 ± 17	NS
Median (range)	8 (2-76)	34 (1-76)	44 (2-47)	41 (3-67)	
ITK use	n = 1 (3%)	0	0	0	

CCH: C-cell hyperplasia; EBRT: external beam radiation therapy; FNAB: fine-needle aspiration biopsy; LN: lymph node; ITK: tyrosine kinase inhibitor; MTC: medullary thyroid cancer; PTC: papillary thyroid cancer; *RET: RE*arranged during *T*ransfection protooncogene; sCt: serum calcitonin; SD: standard deviation; TT: total thyroidectomy; NS: not significant.

a It was considered the most severe clinical presentation (e.g., when patients had metastatic LNs and systemic metastases, it was allocated only into systemic metastases. In some patients, the diagnosis of MTC was discovered many years after TT).

b Not possible to determine in one patient with sporadic MTC because he underwent total thyroidectomy together with total parathyroidectomy for the treatment of hyperparathyroidism caused by parathyroid hyperplasia related to MEN 1 syndrome. Thyroidectomy was performed because of intraoperative suspicion for parathyroid carcinoma. Thyroid tissue histopathology revealed the presence of a microscopic MTC.

c Fisher's exact test among all groups.

d ANOVA test among index patients with hereditary disease, relatives before *RET* screening, and relatives after *RET* screening.

**Figure 5:**
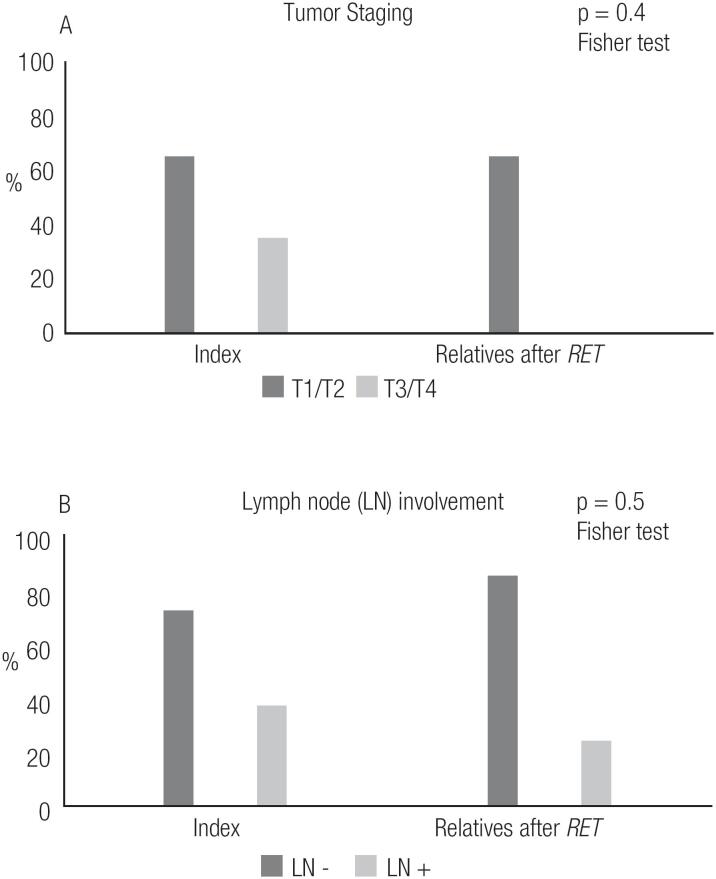
Postoperative staging of the index patients with sporadic and hereditary disease and *RET* mutation-carrier relatives.

Regarding pathologic tumor and LN staging, we observed that relatives whose diagnoses of MTC/CCH were made after *RET* screening had smaller tumor dimensions than index patients (p < 0.05), although there was no significant difference in the frequency of LN involvement between the two groups (Figure 5, Table 4).

**Table 4:** Postoperative staging of the index patients with sporadic and hereditary disease and *RET* mutation-carrier relatives with histopathologically confirmed MTC/CCH after total thyroidectomy

Pathology data	Index		Relatives		p^c^
	Sporadic (n = 31)	Hereditary (n = 28)	Before *RET* screening (n = 6)	After *RET* screening (n = 18)	
**Pathologic tumoral and lymph node staging**	n = 31 (100%)	n = 28 (100%)	n = 6 (100%)	n = 18 (100%)	
CCH only	0	0	0	2
pTx	2	3	1	1	< 0.05
pT1a	12	7	0	9
pT1b	2	4	0	5	
pT2	6	4	2	1
pT3	6	8	3	0	
pT4a	2	2	0	0
pT4b	1	0	0	0	
pNx	13	14	3	4	0.03
pN0	10	2	0	10	
pN1a	0	5	2	1
pN1b	8	7	1	3	
**Multifocality**	n = 27 (87%)	n = 27 (96%)	n = 5 (83%)	n = 15 (94%)	< 0.05
Yes	6	22	4	12^c^	
No	21	5	1	3
Unknown^a^ / Not possible due to large mass^b^	2/ 2	1/ 0	1/0	1/0	
**IHC positive for MTC/CCH**	n = 16 (52%)	n = 27 (96%)	n = 5 (83%)	n = 17 (94%)	NS
Yes	12	18	4	7^d^	
No	4	9	1	10
Not performed	15	1	1	1	
**Association with PTC**	n = 29 (94%)	n = 27 (96%)	n = 5 (83%)	n = 17 (94%)	NS
Yes	3	3	1	2^d^	
No	26	24	4	15
Unknown^a^	2	1	1	1	

a Not possible due to previous partial thyroidectomy and unavailable histopathology results.

b Assessment of multifocality was also not possible in large tumors occupying the entire thyroid. During the initial stage of the disease, it was not possible to determine if there were multiple tumor foci that coalesced or if there was a unique focus that increased in size with time.

c Chi-square test.

*RET: RE*arranged during *T*ransfection protooncogene; CCH: C-cell hyperplasia; IHC: immunohistochemistry; MTC: medullary thyroid cancer; PTC: papillary thyroid cancer; NS: not significant.

A higher proportion of index patients with hereditary disease and their relatives presented tumor multifocality than did index patients with sporadic disease, although the percentage of multifocality was not negligible (22%) in the latter group (Table 4). This finding led us to perform extended *RET* gene analysis in index patients with sporadic disease to exclude the presence of mutations in other exons. However, no patients presented with new mutations in extended *RET* gene analysis.

We observed simultaneous MTC and papillary thyroid carcinoma (PTC) in 9 of 78 (11.5%) patients with MTC (Table 4). In some patients, this association could not be evaluated because these patients had previously undergone a partial thyroidectomy and did not have available histopathology results.

## DISCUSSION

Identification of *RET* mutations in patients with a diagnosis of MTC is essential, both for index patients and for at-risk relatives, and allows for the potential identification of hereditary MTC patients appearing to have sporadic MTC who are not followed-up regarding other MEN 2A components, especially the life-threatening disease PHEO (1). For patients who are already clinically suspected of having hereditary MTC, this analysis allows identification of the specific *RET* mutation and an estimation of the disease's likely clinical course. For affected family members, *RET* mutation analysis allows MTC to be cured when prophylactic thyroidectomy is performed and also allows precocious detection of and administration of therapy for other components of the syndrome. For non-affected family members, it spares these patients from having to undergo clinical and biochemical monitoring for all MEN 2A components (9).

Using clinical history and physical examination findings alone, we identified only 6 hereditary MTC index cases. However, *RET* screening allowed us to identify unsuspected germline *RET* mutations in 23 of the 54 MTC patients (43%) who appeared to present as sporadic disease cases, according to their negative family histories and isolated MTC phenotype (Table 2). Therefore, through *RET* screening, the number of index patients with hereditary MTC increased to 29. Elisei and cols. also found unsuspected germline *RET* mutations in some sporadic cases but at a percentage (only 7.3% of cases) that was much lower than in our study (5). We believe that some particular findings of this study may explain this difference, such as the higher prevalence of the p.M918V mutation (> 30%) among hereditary MTC cases (Figure 2) marked by the isolated MTC phenotype, as well as the higher percentage of hereditary index patients (48%) in this study compared to the 25% usually described in the literature (5,10-12). Regarding this higher percentage of hereditary index patients in Ceará compared to the literature, we believe that this may reflect the high frequency of mutation dispersion in the past, before the existence of birth control policies. Families were larger and comprised many children. In addition, the frequency of extramarital relationships, which has been reported to be a prevalent practice, was higher and may have resulted in an increased number of births outside of marriage (13).

In our initial evaluation, the most common mutation found in Ceará was p.M918V (Figure 2). Thus far, this mutation has been identified in 9 families who were apparently unrelated. Previously, this mutation had been described in only one patient with MTC and in 1 family member without a neoplasm in the literature (14). As the number of index patients who were found to have the p.M918V mutation was increased, we observed that some of those patients lived in nearby regions, such as in the Northeastern region of Ceará State. This finding led us to question whether a founder effect could explain the high frequency of the p.M918V mutation in Ceará, as at that time, 8 families with a p.M918V mutation had already been identified. The evidence suggestive of a founder effect as an explanation for the frequency of the p.M918V mutation in Ceará led us to consider whether the first 8 families diagnosed with a p.M918V mutation were actually one large family (8).

The most frequent mutations identified in our study were in codons 918 and 804 (Figure 2, Table 2), findings that differ from those of other studies worldwide, in which mutations in codon 634 were the most common mutations found (15-27). Also, mutations in codons 338, 515, 666, 618, 620, 768, 848, 883 and 904, which were observed in the European studies (25), have not been identified in Ceará thus far (Table 5).

**Table 5:** Prevalence of *RET* gene mutations in the State of Ceará along with major international studies and their respective genotype-phenotype correlations

*RET* mutations	Exon	ATA risk	MEN 2A	MEN 2B	Ceará, Brazil	ITAMEN (22)	Germany, Halle 1994-2012 (25)	EUROMEN (23)	France, multicentric (21, 23)
					n (%)	n (%)	n (%)	n (%)	n (%)
**p.T338I**	5	MOD	**X**		0	1 (0.4)	0	0	0
**p.C515S**	8	MOD	**X**		0	1 (0.4)	0	0	0
p.C609F/G/**G**/R/S/Y	10	MOD	**X**		1 (3)	6 (2.5)	1 (0.5)	1 (0.7)	1 (1)
p.C611**F**/**G** /R/S/W/Y	10	MOD	**X**		1 (3)	1 (0.4)	6 (3.1)	4 (2.8)	1 (1)
p.C618G/R/**R**//F/S/Y	10	MOD	**X**		0	15 (6.1)	11 (5.8)	10 (7)	6 (6)
p.C620 F/**F**/**G**/R/S/**W**/Y	10	MOD	**X**		0	9 (3.7)	14 (7.3)	10 (7)	12 (12)
p.C630 **F**/R/**R**/**S**/Y	11	MOD	**X**		1 (3)	4 (1.6)	1 (0.5)	1 (0.7)	0
p.C634F/G/R/S/W/Y	11	HIGH	X		4 (14)	86 (35.2)	73 (38.2)	98 (69)	46 (47)
**p.K666M**	11	MOD	**X**		0	1 (0.4)	0	0	0
**p.E768D**	13	MOD	**X**		0	9 (3.7)	2 (1)	1 (0.7)	2 (2)
p.L790F	13	MOD	X		1 (3)	8 (3.3)	26 (13.6)	7 (4.9)	4 (4)
p.V804L/M/**M**	14	MOD	**X**		10 (34)	52 (21.3)	19 (10)	3 (2.1)	15 (15)
p.M848T	14	MOD	**X**		0	1 (0.4)	0	0	0
p.A883T	15	MOD	**X**		0	1 (0.4)	0	0	0
p.S891A/**A**	15	MOD		X	1 (3)	23 (9.4)	6 (3)	3 (2.1)	7 (7)
p.S904F	14	MOD	**X**		0	1 (0.4)	0	0	0
p.M918T	16	HIGHEST	X		1 (3)	17 (7.0)	32 (16.8)	4 (2.8)	3 (3)
p.M918**V**	16	MOD	**X**		9 (31)	2 (0.8)	0	0	0
Without mutations	_	_	**X**		0	6 (2.5)	0	0	0
TOTAL					29 (100)	244	191 (100)	142 (100)	97 (100)

The mutations causing MEN 2A and MEN 2B are shown in black X, whereas the FMTC mutations are shown in bold **X**.

ATA: American Thyroid Association; MTC: medullary thyroid carcinoma; FMTC: familial medullary thyroid carcinoma; MOD: moderate; *RET: RE*arranged during *T*ransfection; MEN: multiple endocrine neoplasia. Modified from references 4 and 25.

Regarding preoperative evaluations, the low sensitivity of FNAB cytology noted in this study (20%) (Figure 3) had previously been reported in other studies (28-30). Essig Jr and cols. concluded that cytological evaluations alone limited one's ability to perform an optimal preoperative assessment and initial surgery in over half of affected patients in sporadic MTC (28).

The present study led us to analyze the potential benefits of diagnosing affected relatives. Comparison of mutation-carrier relatives diagnosed with MTC after *RET* screening to index patients showed that those relatives had a lower frequency of complications, such as post-surgical hypoparathyroidism, and lower levels of sCt upon their last visit (p < 0.05). Also, none of the affected relatives died, while 5 indexes succumbed to disease (Figure 4). Interestingly, the affected relatives were not diagnosed at a significantly younger age (Table 3), nor were they diagnosed at a more favorable clinical or pathologic stage than their counterparts (Table 4). In fact, a trend toward diagnosis at a younger age was observed, but the trend was not statistically significant (Table 3). We also observed that relatives who had a diagnosis of MTC after *RET* screening had smaller tumor dimensions than index patients, but the degree of lymph node involvement was similar between the two groups (Figure 5). In fact, 50% of those mutation-carrier relatives diagnosed with MTC after *RET* screening already presented clinical evidence of MTC before the *RET* screening results were available (Table 1). Therefore, in our study population, the benefits of *RET* screening were not fully realized. We consider that the major reason for this was because these patients received this exam relatively late, as they were not aware of its existence and benefits. We believe that if we can offer earlier *RET* screening for at-risk patients, we will be able to diagnose neoplasms at an earlier stage, or, more desirably, before they have developed.

The finding of simultaneous MTC and PTC in this study (11.5%) had previously been described in previous studies and was observed in the following variable percentages of patients in those studies: 3.6% of patients in Germany (31), 13.8% of patients in Italy (32) and 19% of patients in Korea (33). Those discrepancies were attributed to environmental conditions and differences in the populations of the studies in question (1).

Regarding tumor multifocality, this phenomenon was observed more frequently in index patients with hereditary disease and their relatives than in index patients with sporadic disease. Consistent with the findings of the study by Lindsey and cols. (7), in the present study, we noted no direct clinical benefit to extending the *RET* germline analysis beyond the hot-spot regions in patients with sporadic MTC with multifocality (22%) since the analysis did not identify any mutations in other exons. Therefore, these cases were true sporadic cases of MTC with multifocal tumors, which had been observed in a recent study (34).

Our study population comprised a significant number of mutation carriers who did not attend follow-up visits after learning their *RET* sequencing results, nor did they bring their at-risk relatives to a clinic to undergo *RET* screening. Geographic distances and some patients’ poor financial statuses, which prevented them from bearing the financial burden imposed by traveling to our center, are recognized as important factors that delayed the performance of *RET* screening. We also considered other reasons that may explain some patients’ delays in undergoing genetic analysis and some patients’ refusal to undergo genetic analysis. First, not every *RET* gene carrier is emotionally prepared to face a diagnosis of MTC or to share personal genetic information with his or her relatives. These types of behavior may be related to feelings of guilt and resentment, emotional distress, and poor familial interactions (25,35). Other obstacles affecting patients’ abilities to undergo genetic analysis are related to individual aspects, such as patients’ understanding of their disease and religious beliefs. In contrast to the above findings, in some families, we observed solidarity among family members. Additionally, we noted that some family members felt a need to reveal their diagnosis and their intention to face their disease positively in order to motivate their relatives to accelerate their diagnostic and treatment processes to avoid being diagnosed with a neoplasm at the same stage that they had been.

In conclusion, in our study, *RET* screening was profoundly crucial for the early diagnosis of MTC and facilitated monitoring of other MEN 2A components and identification of unsuspected germline mutations in index patients with apparent sporadic disease. We noted a mutation profile that differed from that of previous European studies. We attribute these differences to our having restricted our analysis to the State of Ceará and to our having evaluated a small number of patients. These features make the extrapolation of our results to the rest of the country unlikely, as different colonization patterns occurred throughout Brazil. However, we believe that any regional differences in *RET* distributions that exist are likely to diminish as a result of dynamic world social, political and economic scenarios impacting migration processes.
